# Rapid antiretroviral therapy and treatment outcomes among people living with HIV: exploring the mediating roles of medication adherence

**DOI:** 10.3389/fpubh.2024.1420609

**Published:** 2024-10-01

**Authors:** Hao Chen, Ran Tao, Lingli Wu, Cheng Chen, Jingchun He

**Affiliations:** Center for Disease Control and Prevention of Jiulongpo District, Chongqing, China

**Keywords:** HIV, rapid ART initiation, medication adherence, viral failure, mediation effect

## Abstract

**Introduction:**

The rapid initiation of antiretroviral therapy (ART) and its impact on treatment outcomes have been a subject of global public health interest. However, the precise mechanisms underlying the effects of rapid ART initiation remain unclear.

**Methods:**

This retrospective cohort study examined data from 1846 HIV-infected individuals in Jiulongpo District, Chongqing, China, spanning from 2016 to 2022. Logistic regression models and serial mediation analysis were used to explore the influence of rapid ART initiation on treatment outcomes and the role of medication adherence as a mediating factor.

**Results:**

The findings revealed a significant association between rapid ART initiation and reduced risk of viral failure (adjusted odds ratio [OR] = 0.320, 95% confidence interval [CI] = [0.161, 0.637]), as well as an increased likelihood of improved adherence (adjusted OR = 2.053, 95% CI = [1.226, 3.438]). Medication adherence was identified as a partial mediator in the relationship between rapid ART initiation and viral failure, explaining 10.5% of the total effect.

**Discussion:**

In conclusion,rapid initiation of antiretroviral therapy was found to enhance treatment outcomes, emphasizing the importance of early adherence education. The study recommends early initiation of ART coupled with adherence education and psychological counseling for HIV-infected individuals.

## Introduction

HIV/AIDS remains a critical global public health challenge and a predominant cause of mortality worldwide. In 2022, there were 39 million people globally living with HIV/AIDS, with 1.3 million new HIV infections, and 630,000 deaths attributed to HIV/AIDS-related illnesses (1). Despite the HIV epidemic has largely improved since the introduction of antiretroviral therapy (ART) (2), ongoing efforts to achieve the goal of ending AIDS require continuous adaptation of treatment protocols. Initially, the initiation of ART for people living with HIV (PLWH) relied on CD4 cell count thresholds (3). Subsequently, the World Health Organization (WHO) issued the “treat all” policy, advocating for HIV treatment regardless of CD4 cell count or clinical symptoms (4). Presently, the WHO recommends the rapid initiation of antiretroviral therapy (Rapid ART), which defined as the initiation of ART within 7 days of diagnosis and recommends that prepared individuals should begin treatment on the same day of diagnosis (5).

The mechanism by which rapid ART enhances treatment outcomes may be associated with the reduction of the viral reservoir. Clones of infected cells can arise as early as 20 days after HIV infection (6), and a viral reservoir of long-lived, transcriptionally silent cells is established very early in the peripheral blood, lymphoid tissue, lungs, brain, spleen, and gut (7). This reservoir remains despite ART and leads to viral rebound when ART is discontinued. While rapid ART can reduce the plasma viral load, lower the viral set point, and limit the size of the latent HIV reservoir (8–10). Additionally, Lee et al. found very limited identical viral sequences after early ART initiation, which might imply that there is little clonal proliferation of CD4+ T-cells, an important mechanism fueling persistence of the HIV reservoir (11).

Numbers of evidence suggest that the rapid initiation of treatment can yield both clinical and public health benefits. Rapid ART has been demonstrated to increase ART initiation rates (12, 13), shorten the time to achieve viral suppression (13–16), reduce the risk of HIV transmission (12, 17, 18), decrease morbidity and mortality (19, 20), and mitigate patient loss to follow-up (17, 21). Furthermore, it has shown potential in decreasing the incidence of tuberculosis and severe bacterial infections (22). Epidemiological and economic analyses conducted in Spain have indicated that rapid ART initiation could prevent approximately 992 potential HIV infections and result in an estimated €323 million in potential savings over the next two decades (23).

Treatment adherence is a backbone for the success of ART (24). A retrospective cohort study conducted in Uganda demonstrates that individuals with better adherence are more likely to achieve viral suppression (24). Additional research suggests poor adherence is associated with ART failure and the occurrence of drug resistance (25, 26). Furthermore, adherence appears to be a crucial factor in the relationship between rapid ART and treatment outcomes. On the one hand, rapid ART can reduce treatment loss (27) and maintain treatment retention (12); on the other hand, hasty rapid ART may decrease willingness to be treated (5). However, there is limited research in real-world settings on the mechanisms linking adherence to rapid ART and treatment outcomes.

In China, the comprehensive implementation of the “treat all” policy began in 2016, laying the groundwork for rapid ART, which commenced in 2021. The purpose of this study is to (1) explore the association between rapid ART and viral suppression in real world, and (2) investigate the role of medication adherence among these two factors.

## Materials and methods

### Data source and collection

This observational retrospective study examined a cohort of patients diagnosed with HIV from January 2016 to December 2022. Data were sourced from the China Information System for Disease Control and Prevention, which record diagnosis and treatment details through reporting agencies and designated treatment facilities. This study was conducted in the Jiulongpo District of Chongqing, where three government-designated healthcare institutions (Chongqing Public Health Medical Center (CMC), The First Public Hospital in Jiulongpo (FPH) and The Second Public Hospital in Jiulongpo (SPH)) provided antiretroviral therapy services for HIV/AIDS and authorized healthcare personnel inputted information of PLWH, including personal, treatment, and epidemiological data.

### Study population

**Figure 1:**
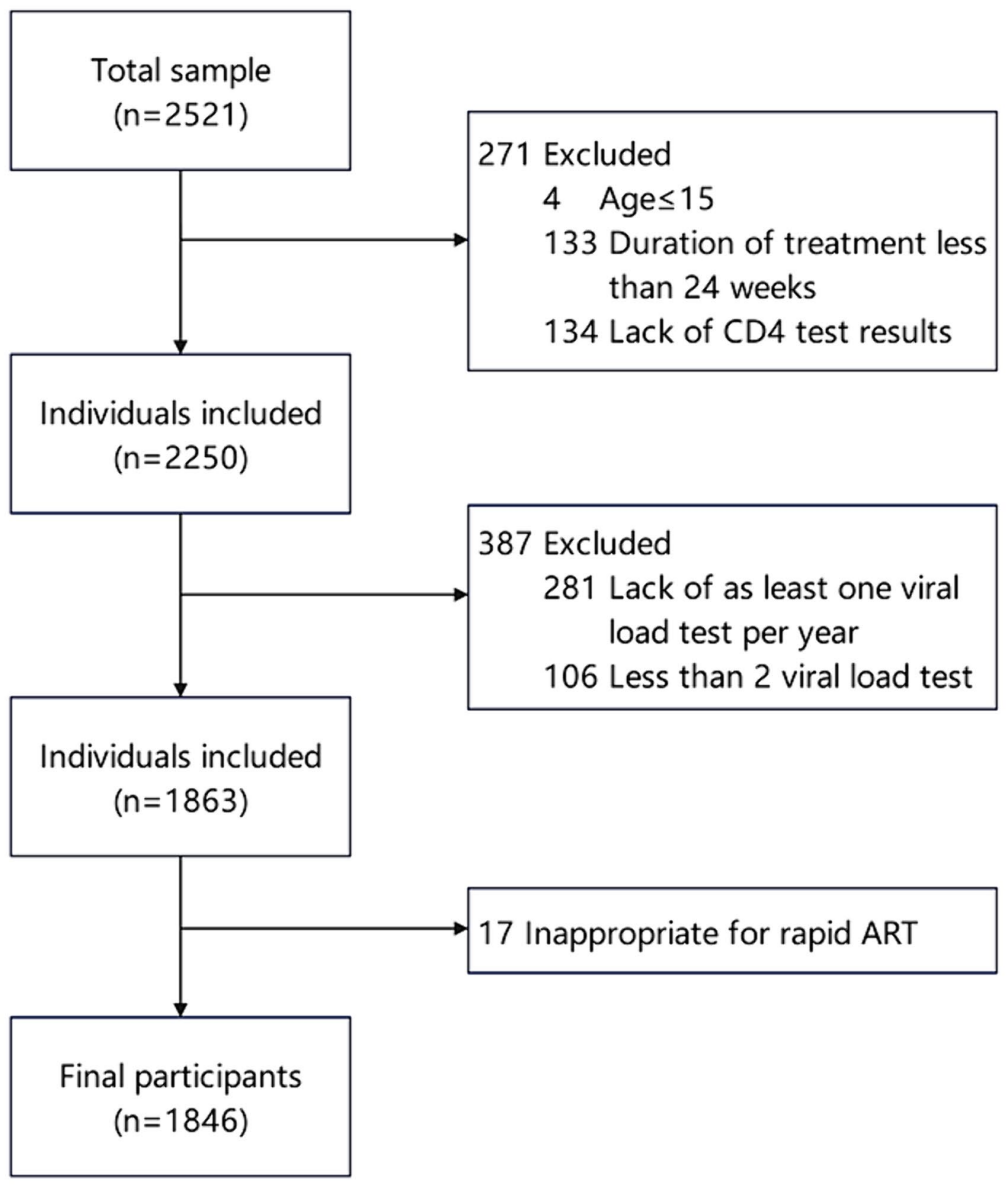
Flow of participant inclusion among PLWH initiating antiretroviral therapy in Jiulongpo District from 2016 to 2022.

We included patients who registered for HIV diagnosis and treatment in the information system and screened the study population based on the following criteria. Inclusion criteria: (1) Current address was in Jiulongpo District of Chongqing; (2) ART initiation occurred between January 2016 and December 2022. Exclusion criteria: (1) Age < =15; (2) Duration of ART less than 24 weeks; (3) Patients without CD4 test results before ART initiation; (4) Absence of at least one viral load test per year after 24 weeks of ART, with a total number of viral load tests less than two; (5) Indications of inappropriateness for rapid ART, including pulmonary tuberculosis, extrapulmonary tuberculosis, mycobacterium avium complex infection, cryptococcal meningitis, serious chronic diseases, hepatic and renal insufficiency, nervous system diseases, pneumocystis carinii pneumonia, Penicillium marneffei. The filter process is shown in Figure 1.

### Measures

Outcome-The outcome of interest was viral failure (VF), defined dichotomously as two consecutive plasma HIV-1 RNA measurements exceeding 200 copies/ml after 24 weeks of ART, according to the latest manual on free antiviral drug treatment in China (2023), as opposed to viral suppression (*VS*). An increasing number of studies utilized continuous measurements of viral load to define VF (28, 29).

Predictor-The predictor of interest was rapid ART, defined as the initiation of ART within 30 days of diagnosis. Currently, there is no unified consensus on the timing of treatment initiation, and the Chinese Guidelines for Diagnosis and Treatment of Human Immunodeficiency Virus Infection /Acquired Immunodeficiency Syndrome (2021 edition) do not provide specific regulations regarding the initiation timing for confirmed cases. Furthermore, due to the relatively late introduction of Rapid ART in China, with only 18.7% of patients starting ART within 7 days (30), we defined rapid ART initiation as treatment initiation within 30 days to align with some nationwide studies (20, 27).

Mediators-The focus mediator was medication adherence, determined by the adherence rate calculated as the number of days medication was taken divided by the prescribed number of days. Adherence was classified into two groups: optimal medication adherence (≥95% adherence) and poor medication adherence (<95% adherence) (31, 32).

Covariates-Covariates were chosen *a priori* based on their potential for confounding. Covariates included sex, age, marital status, CD4 cells count, occurrence of related symptoms, WHO clinical stage, ART regimen, and duration of ART at baseline. Age was dichotomized into two groups: <50 years old and ≥ 50 years old. ART regimen are categorized into first-line, second-line, and others. The first-line treatment includes two nucleoside reverse transcriptase inhibitors (NRTIs) and one non-nucleoside reverse transcriptase inhibitor (NNRTI). The second-line treatment substitutes the NNRTI with either an integrase strand transfer inhibitor (INSTI) or a protease inhibitor (PI).

### Analyses

Descriptive analyses were conducted to summarize participant characteristics. To test for differences, we used chi-square or Fisher test for independence for categorical variables and Mann–Whitney U test for continuous variables.

To evaluate the relationship between rapid ART and VF, logistic regression was used to estimate the total effect of rapid ART on VF. In these models, we initially included only rapid ART to estimate the crude odds ratios (COR), then all covariates were adjusted to calculate adjusted odds ratios (AOR). Furthermore, to explore the roles of medication adherence as mediators, we estimated indirect and direct effects along possible pathways using mediation analysis with 95% confidence intervals (95%CI), generated from bias-corrected bootstrapped standard errors.

All analyses were conducted using R4.1.0. Statistical significance was considered at *p* < 0.05.

## Results

### Participant characteristics

From 2016 to 2022, a total of 2,521 people living with HIV (PLWH) initiated antiretroviral therapy, and finally, 1846 of them were included in the present study (Figure 1). The participant characteristics of the included PLWH are presented in Table 1. The majority of PLWH were male (77.2%), with 39.4% of participants aged 50 and above, and 44.5% married.

**Table 1:** Baseline characteristics of patients and the differences between viral failure and viral suppression.

Variables	Total (*n* = 1,846)	*VS* (*n* = 1,807)	VF (*n* = 39)	*P*	Z/χ^2^
Sex, *n* (%)				0.967	0.002
Male	1,425 (77.2)	1,395 (77.2)	30 (76.9)		
Female	421 (22.8)	412 (22.8)	9 (23.1)		
Marital status, *n* (%)				0.692	0.736
Single	703 (38.1)	690 (38.2)	13 (33.3)		
Married or cohabiting	822 (44.5)	802 (44.4)	20 (51.3)		
Divorced, widowed or unknown	321 (17.4)	315 (17.4)	6 (15.4)		
Occurrence of related symptoms, *n* (%)				0.557	0.345
No	1734 (93.9)	1,696 (93.9)	38 (97.4)		
Yes	112 (6.1)	111 (6.1)	1 (2.6)		
WHO clinical stage, *n* (%)				0.069	
Stage 1	385 (20.9)	379 (21)	6 (15.4)		
Stage 2	1,125 (60.9)	1,105 (61.2)	20 (51.3)		
Stage 3	115 (6.2)	112 (6.2)	3 (7.7)		
Stage 4	221 (12)	211 (11.7)	10 (25.6)		
Adherence, *n* (%)				< 0.001	51.623
Optimal	1779 (96.4)	1751 (96.9)	28 (73.7)		
Poor	66 (3.6)	56 (3.1)	10 (26.3)		
Baseline CD4 count, cells/μl, Median (Q1, Q3)	207 (116.25, 318)	210 (120, 319.5)	123 (47, 218.5)	< 0.001	−3.659
Duration on ART, Median (Q1, Q3)	1,296 (766, 1887.75)	1,293 (759, 1885.5)	1,642 (1,152, 1930)	0.009	−2.625
Age group, *n* (%)				0.9	0.016
<50	1,118 (60.6)	1,094 (60.5)	24 (61.5)		
≥50	728 (39.4)	713 (39.5)	15 (38.5)		
Initiation time of ART, *n* (%)				0.002	10.001
Rapid ART(<30d)	1,115 (60.4)	1,101 (60.9)	14 (35.9)		
Delayed ART(≥30d)	731 (39.6)	706 (39.1)	25 (64.1)		
ART regimen, *n* (%)				0.064	
First-line treatments	1,496 (81.0)	1,459 (80.7)	37 (94.9)		
Second-line treatments	91 (4.9)	90 (5)	1 (2.6)		
Others	259 (14.0)	258 (14.3)	1 (2.6)		

In terms of baseline clinical presentation, the majority of individuals (93.9%) had no related symptoms, with over half (60.9%) being at WHO clinical stage 2. Additionally, the median CD4 count prior to treatment initiation was only 207, and during the treatment process.

Regarding treatment aspects, the majority of individuals (81.0%) were prescribed first-line treatment regimens, with 96.4% exhibiting optimal medication adherence. The median duration of treatment was 1,296 days, and 60.4% of PLWH initiated treatment within 30 days.

The total viral failure rate was 2.11%. Compared to cases with *VS*, patients experiencing VF appeared to have poor medication adherence, lower baseline CD4 counts, longer treatment durations, and a lower proportion of rapid ART initiation (all *p* < 0.05).

### Effect of rapid ART on vial failure

In the crude model, rapid ART had a significantly association with VF, such that rapid ART was associated with lower odds of VF (COR = 0.359, 95% CI = [0.185, 0.696]). These results were similar with those of the fully adjusted model. Specifically, rapid ART was associated with a 68% decrease in the odds of VF (AOR = 0.320, 95% CI = [0.161, 0.637]). In addition, there was an association between VF and baseline CD4 levels, with odds ratios and 95% CI of 0.995 (0.992, 0.998), as showed in Table 2.

**Table 2:** The association between rapid ART and viral failure.

Variables	Model 1		Model 2	
	COR (95%CI)	*P*	AOR (95%CI)	*P*
Initiation time of ART				
Delayed ART(≥30d)	1		1	
Rapid ART(≤30d)	0.359 (0.185–0.696)	0.002	0.320 (0.161–0.637)	0.001
Sex				
Male			1	
Female			0.935 (0.42–2.078)	0.868
Marital status				
Single			1	
Married or cohabiting			1.218 (0.521–2.848)	0.650
Divorced, widowed or unknown			0.892 (0.298–2.676)	0.839
Weather occur HIV-related diseases				
No			1	
Yes			0.217 (0.028–1.679)	0.143
WHO clinical stage				
WHO clinical stage 1			1	
WHO clinical stage 2			1.71 (0.611–4.787)	0.307
WHO clinical stage 3			2.042 (0.47–8.883)	0.341
WHO clinical stage 4			3.133 (0.984–9.976)	0.053
ART regimen				
First-line treatments			1	
Second-line treatments			0.505 (0.066–3.851)	0.510
Others			0.268 (0.034–2.097)	0.210
Age group				
Age < 50			1	
Age ≥ 50			0.894 (0.411–1.945)	0.778
Baseline CD4 count			0.995 (0.992–0.998)	0.002
Duration on ART			1.001 (1–1.001)	0.054

COR: crude odds ratios; AOR: adjusted odds ratios; 95%CI: 95% confidence interval. Model 1: Crude model with no covariates were adjusted. Model 2: Fully adjusted model with adjusted for sex, age, marital status, CD4 cells count, occurrence of HIV-related diseases, WHO clinical stage, ART regimen, and duration of ART at baseline.

### Effect of rapid ART on adherence

The results indicated that, without adjusting for confounding factors, rapid ART could significantly increase medication adherence (COR = 1.761, 95% CI = [1.076, 2.882]). Correspondingly, after controlling for all confounding variables, the AOR for rapid ART improving adherence was 2.053 (95% CI = [1.226, 3.438]). Additionally, WHO stage 4 was associated with decreased adherence (AOR = 0.195, 95% CI = [0.064, 0.592]), as showed in Table 3.

**Table 3:** The association between initiation time of ART and medication adherence.

	Model 1		Model 2	
Variables	COR (95%CI)	*P*	AOR (95%CI)	*P*
Initiation time of ART				
Delayed ART(≥30d)	1		1	
Rapid ART(≤30d)	1.761 (1.076–2.882)	0.024	2.053 (1.226–3.438)	0.006
Sex				
Male			1	
Female			1.068 (0.579–1.969)	0.834
Marital status				
Single			1	
Married or cohabiting			0.836 (0.404–1.733)	0.630
Divorced, widowed or unknown			0.577 (0.257–1.296)	0.183
Weather occur HIV-related diseases				
No			1	
Yes			1.105 (0.4–3.055)	0.848
WHO clinical stage				
WHO clinical stage 1			1	
WHO clinical stage 2			0.48 (0.187–1.233)	0.127
WHO clinical stage 3			0.395 (0.103–1.515)	0.176
WHO clinical stage 4			0.195 (0.064–0.592)	0.004
ART regimen				
First-line treatments			1	
Second-line treatments			0.704 (0.266–1.863)	0.480
Others			2.158 (0.86–5.413)	0.101
Age group				
Age < 50			1	
Age ≥ 50			0.682 (0.368–1.264)	0.224
Baseline CD4 count			0.999 (0.997–1.001)	0.209
Duration on ART			1 (1–1.001)	0.140

COR: crude odds ratios; AOR: adjusted odds ratios. Model 1: Crude model with no covariates were adjusted. Model 2: Fully adjusted model with adjusted for sex, age, marital status, CD4 cells count, occurrence of HIV-related diseases, WHO clinical stage, ART regimen, and duration of ART at baseline.

### The moderate effect of adherence

**Figure 2:**
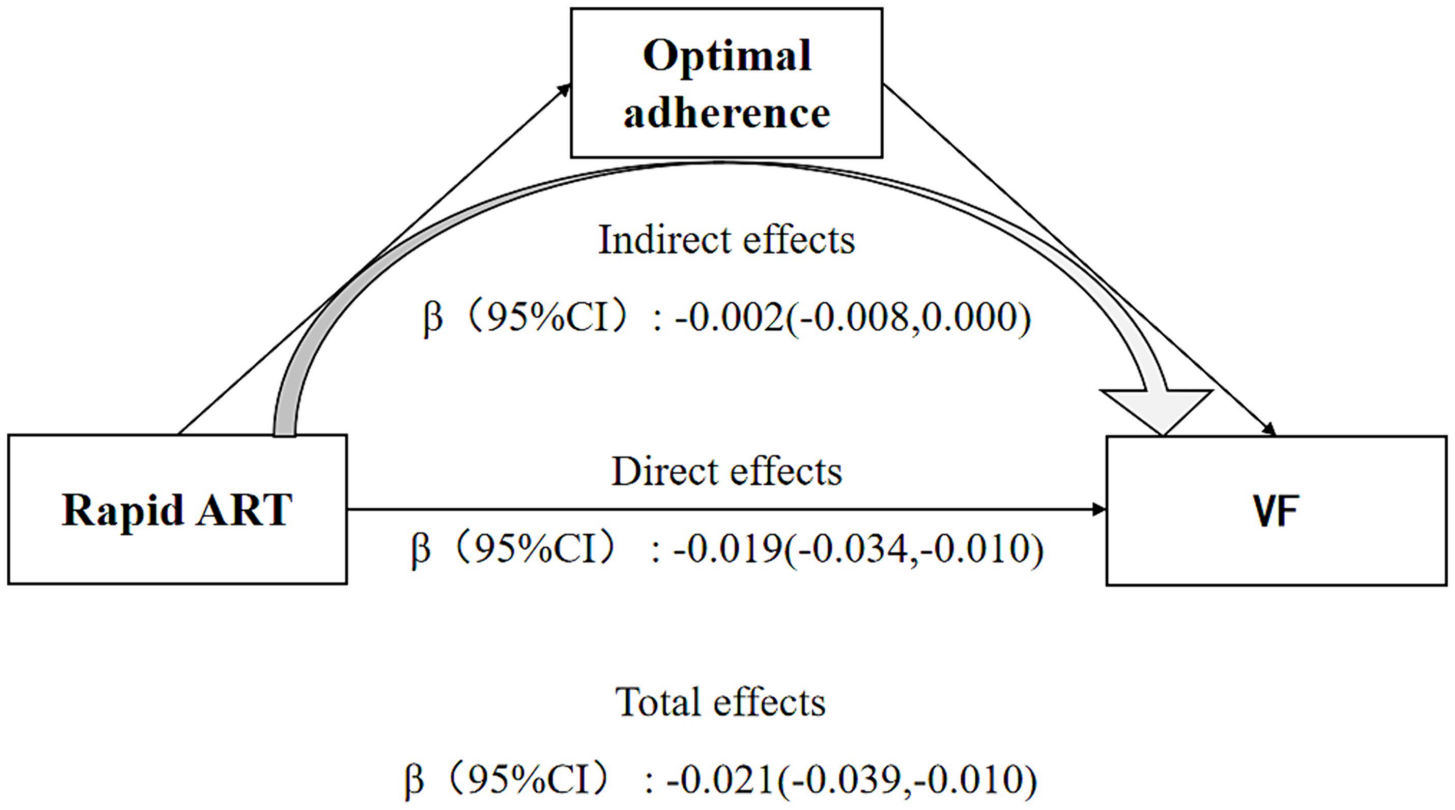
Direct and indirect effects of rapid ART on viral failure depending on the moderator medication adherence. Sex, age, marital status, CD4 cells count, occurrence of HIV-related diseases, WHO clinical stage, ART regimen, and duration of ART at baseline were controlled in the model.

Figure 2 displayed the findings of serial mediation analyses, presenting total effects as differences in log-odds for comparison. After accounting for potential mediators, rapid ART was found to exert a statistically significant negative direct effect on VF (*β* = −0.019, 95% CI = [−0.034, −0.01]), and this direct effect was of lesser magnitude compared to the estimated total effect of rapid ART (*β* = −0.021, 95% CI = [−0.039, −0.01]).

The indirect effect of Rapid ART on reducing the risk of VF through enhancing medication adherence was also significant (*β* = −0.002, 95% CI = [−0.008, 0.000]). 10.5% of the total effect could be attributed to the mediating role of adherence.

## Discussion

In present study, nearly 60% of participants initiated rapid ART, which was lower than the data from a recent study conducted in China, where the ART initiation rate within 30 days for newly diagnosed HIV-infected individuals in China was reported to be close to 75% (33). Furthermore, individuals initiating rapid ART were more likely to exhibit optimal medication adherence and lower risk of viral failure. Mediation analysis revealed that medication adherence partially mediated the association between rapid ART initiation and viral failure.

In this study, we found a significant association between rapid ART and a lower likelihood of viral failure, even when adjusting for potential confounders. This discovery was consistent with findings from previous studies involving PLWH. For instance, A large retrospective observational cohort study conducted in China found that patients who received immediate ART (within 30 days of diagnosis) had a lower rate of viral failure compared to those who initiated ART later (27). In a prospective randomized controlled trial, the viral suppression rate at 12 months of treatment was 50.4% in the rapid initiation group, compared to only 34.3% in the standard protocol group (12). And another comprehensive retrospective analysis of newly diagnosed PLWH in high-income countries has demonstrated that immediate initiation of antiretroviral therapy (ART) accelerates viral suppression during follow-up (34).

Some studies suggested that rapid initiation of treatment might lead to decreased adherence, which contradicted our research findings (31, 35). This discrepancy could be attributed to the definition of rapid initiation treatment as initiating treatment on the same day of diagnosis in their studies, whereas PLWH might not psychologically accept the fact of infection in a short period of time and might harbor biases against treatment due to lack of information about antiretroviral therapy. In contrast, a retrospective cohort study conducted in China suggested that rapid ART within 7 days may actually improve adherence (36). This suggests that newly diagnosed patients require a certain degree of buffering before initiating treatment.

Numerous studies had demonstrated that medication adherence is a key influencing factor for the success of antiviral therapy (24, 37), yet few explored the role of adherence in the causal pathway between rapid treatment initiation and treatment outcomes. This study indicated that good adherence served as a mechanism for the reduction of viral failure risk associated with rapid ART, with the mediating effect accounting for 10.5%. Although more and more drugs are now being used for HIV treatment, some of which have a high genetic barrier to maintain a good virological response, a large proportion of patients are still on previous regimens. Therefore, to enhance the effectiveness of rapid treatment initiation, it is imperative to strengthen education on communication methods for healthcare professionals, and this will enable them to accurately and effectively convey the benefits of rapid treatment initiation to New HIV-infected individuals within limited time frames. Additionally, providing psychological counseling for patients can help improve their acceptance and adherence to treatment.

This study also has some limitations. Firstly, only a small proportion of patients underwent viral load testing before treatment initiation, thus the confounding effect of baseline viral load could not be controlled. Additionally, there were other potential confounding factors that were not documented systematically, such as alcohol consumption and AIDS related knowledge. Secondly, this study was conducted in one district and the same participants could not represent all PLWH in China; however, it is possible that an even larger sample and multi-center approach may have been necessary.

## Conclusion

In summary, during the study period, the rate of rapid ART was 60.4%, and those who experience rapid ART are more likely to exhibit good adherence and less likely to be VF, indicating that rapid treatment initiation continues to be a crucial strategy. Additionally, improving medication adherence is a partial pathway through which rapid treatment initiation reduces the risk of viral failure. Therefore, this study suggests that adherence counseling should be particularly emphasized for individuals starting ART. Capacity building of healthcare workers in adherence counseling and deploying counselors to ART clinics could help improve adherence and consequently enhance viral load suppression.

## Data Availability

The raw data supporting the conclusions of this article will be made available by the authors, without undue reservation.
